# Cerebrospinal fluid proteomics implicates the granin family in Parkinson’s disease

**DOI:** 10.1038/s41598-020-59414-4

**Published:** 2020-02-12

**Authors:** Melissa S. Rotunno, Monica Lane, Wenfei Zhang, Pavlina Wolf, Petra Oliva, Catherine Viel, Anne-Marie Wills, Roy N. Alcalay, Clemens R. Scherzer, Lamya S. Shihabuddin, Kate Zhang, S. Pablo Sardi

**Affiliations:** 1 Rare and Neurologic Diseases Therapeutic Area, Sanofi, Inc., Framingham, MA 01701 USA; 2 Biomarkers and Bioanalytics, Translational Sciences, Sanofi, Inc., Framingham, MA 01701 USA; 3 Translational Medicine, Sanofi, Inc., Framingham, MA 01701 USA; 4 Precision Neurology Program, Harvard Medical School, Brigham & Women’s Hospital, Boston, MA 02115 USA; 5 Department of Neurology, Columbia University, New York, NY 10032-3784 USA; 6 Department of Neurology, Massachusetts General Hospital, Boston, MA 02114 USA; 7 APDA Center for Advance Parkinson Research, Harvard Medical School, Brigham & Women’s Hospital, Boston, MA 02115 USA; 8 Present Address: Editas Medicine, Cambridge, MA 02141 USA; 9 Present Address: ARCHIMED Life Sciences GmbH, Leberstraße 20/2, 1110 Vienna, Austria

**Keywords:** Neurodegeneration, Parkinson's disease

## Abstract

Parkinson’s disease, the most common age-related movement disorder, is a progressive neurodegenerative disease with unclear etiology. Better understanding of the underlying disease mechanism(s) is an urgent need for the development of disease-modifying therapeutics. Limited studies have been performed in large patient cohorts to identify protein alterations in cerebrospinal fluid (CSF), a proximal site to pathology. We set out to identify disease-relevant protein changes in CSF to gain insights into the etiology of Parkinson’s disease and potentially assist in disease biomarker identification. In this study, we used liquid chromatography-tandem mass spectrometry in data-independent acquisition (DIA) mode to identify Parkinson’s-relevant biomarkers in cerebrospinal fluid. We quantified 341 protein groups in two independent cohorts (n = 196) and a longitudinal cohort (n = 105 samples, representing 40 patients) consisting of Parkinson’s disease and healthy control samples from three different sources. A first cohort of 53 Parkinson’s disease and 72 control samples was analyzed, identifying 53 proteins with significant changes (p < 0.05) in Parkinson’s disease relative to healthy control. We established a biomarker signature and multiple protein ratios that differentiate Parkinson’s disease from healthy controls and validated these results in an independent cohort. The second cohort included 28 Parkinson’s disease and 43 control samples. Independent analysis of these samples identified 41 proteins with significant changes. Evaluation of the overlapping changes between the two cohorts identified 13 proteins with consistent and significant changes (p < 0.05). Importantly, we found the extended granin family proteins as reduced in disease, suggesting a potential common mechanism for the biological reduction in monoamine neurotransmission in Parkinson’s patients. Our study identifies several novel protein changes in Parkinson’s disease cerebrospinal fluid that may be exploited for understanding etiology of disease and for biomarker development.

## Introduction

Parkinson’s disease (PD) is the second most frequent neurodegenerative disease affecting approximately 1 million people in the US. Although the etiology of PD is largely unknown, genetic and environmental factors have been implicated in disease development. The pathological hallmarks of PD are dopaminergic neuronal loss in the substantia nigra pars compacta and presence of Lewy bodies containing aggregated α-synuclein^1–3^. While there are medications to improve motor function in PD, there are no FDA approved interventions to slow down or reverse the disease because of our limited understanding of disease pathogenesis^2–4^. Identifying protein alterations present in PD could provide insight into the underlying mechanism of disease and aid in the development of future novel therapeutics.

Another major challenge of disease modification trials is the lack of objective measurements of disease progression. Current biomarkers are clinical assessments, which are of limited use because of high variability in disease progression, the effect of symptomatic medications on the exam and subjective clinical scoring. The present study set out to identify biomolecular signatures in cerebrospinal fluid (CSF), which could also assist the analysis of therapeutic efficacy and disease modifications in proof of concept trials. The potential utility of CSF biomarkers for neurodegenerative diseases was previously demonstrated by the development of various assays that are currently used for Alzheimer’s disease (*e*.*g*. total Aβ42 and phosphorylated tau^5,6^.

Quantitative proteomics has made significant progress with the development of liquid chromatography-tandem mass spectrometry (LC/MS/MS) in data-independent acquisition mode (DIA-MS)^7^. DIA-MS provides a suitable compromise of sensitivity, reproducibility, and extent of peptide identification, and is mainly limited by the breadth and quality of the protein library that is required in the data analysis pipeline. While traditional proteomic approaches lack reproducible peptide fragmentation and quantification of lower abundant proteins, DIA-MS allows the quantification of the same set of proteins measured in all samples as long as it is above the lower limit of detection and present in the peptide spectral library used in the data analysis pipeline. We selected the PD-relevant biofluid CSF for identifying novel protein changes. Analysis of the CSF proteome presents a unique set of challenges to overcome when interpreting the data output. Simple normalization of protein quantification to CSF volume, for example, adds analytical error, as the total protein concentration varies 3-fold between subjects, ranging from 0.15 to 0.5 mg/ml^8^. Additionally, total protein normalization mainly reflects the concentration of the most abundant proteins, albumin and cystatin-C, both of which are reportedly altered in PD^9–12^. DIA-MS permits the quantification of hundreds of proteins in a single run, allowing for a normalization approach that considers the intensities of all detectable proteins with a more evenly weighted distribution^13^. Considering these advantages and disadvantages, we initially focused on DIA-MS to obtain the best compromise between sensitivity, reproducibility, and extent of peptide identification for quantifying the CSF proteome.

In the present study, we set out to identify protein alterations in PD by utilizing DIA-MS to analyze CSF samples in two independent cohorts consisting of PD and healthy control (HC) subjects. We developed a CSF processing pipeline with reproducible protein quantification and minimal CSF input. Several novel protein aberrations were identified. The changes implicate altered production/processing of biologically active peptides in the extended granin family, cell adhesion, and insulin regulation. Additionally, by employing univariate analyses, LASSO regression and multivariate analyses, we identified a unique biomarker signature and protein ratios that separate PD from HC in both cohorts.

## Methods

### Subjects and sample collection

We analyzed samples collected from 196 participants including 115 HC and 81 PD recruited from three separate sources (Table 1). All subjects provided their gender, age, date of birth, and ethnic background. Patients underwent cognitive function assessment (MMSE: mini-mental state examination; or MoCA: Montreal Cognitive Assessment) and evaluation of disease progression by both the unified Parkinson’s disease rating scale (UPDRS) and Hoehn and Yahr (HY) scoring, except where noted. Principal component analysis did not reveal any batch effect based on CSF source (Additional File 1: Fig. S1**)**.

**Table 1:** Patient demographics and clinical characteristics of all cohorts.

	Cohort 1		Cohort 2		Longitudinal cohort	
Group (Sample **#)**	HC (n = 72)	PD (n = 53)	HC (n = 43)	PD (n = 28)	HC (n = 60)	PD (n = 45)
Gender **(m/f)**	49/23	40/13	27/16	19/9	44/16	27/18
Age (mean ± SD, range)	68 ± 9 (45–83)	67 ± 9 (43–84)	58 ± 10 (36–81)	64 ± 6 (41–72)	56.6 ± 10.4 (35–81)	63.2 ± 8.8 (41–75)
Disease Duration (mean ± SD, range)	—	5.5 ± 4.5 (0–20)	—	4.3 ± 3.3 (0–11)	—	3.8 ± 2.4 (0–9)
HY score^a^ (mean ± SD, range)	—	2.2 ± 0.5 (1–4)*	—	2.1 ± 0.6 (1–4)	—	2.2 ± 0.4 (2–3)
MMSE^b^ (mean ± SD, range)	29.1 ± 1.2 (26–30)	27.4 ± 4.0 (14–30)	27.8 ± 1.4^c^ (25–30)	28.0 ± 2.1^c^ (23–30)	28.7 ± 1.3 (25–30)	29.0 ± 1.2 (26–30)
UPDRS total^d^ (mean ± SD, range)	—	37.6 ± 14.8 (15–73)**	—	41.2 ± 16.5 (19–98)	—	40.5 ± 14.0 (15–73)***
# of Individual Patients	72	53	43	28	22	18
% l-dopa or agonist	—	91%	—	96%	—	94%
Source^e^	HBS = 53; PM = 19	HBS = 35; PM = 18	HBS = 26; CU = 17	HBS = 8; CU = 20	HBS = 60	HBS = 45

*Data not available for five patient samples in this cohort.

**Data not available for nineteen patient samples in this cohort.

***Data not available for one time point for one patient sample.

^a^HY score, Hoehn and Yahr scale.

^b^MMSE, mini-mental state examination.

^c^The samples from Columbia University underwent the Montreal Cognitive Assessment (MoCA), not MMSE.

^d^UPDRS total, unified Parkinson’s disease rating scale.

^e^Harvard biomarkers study, HBS; Columbia University, CU; PrecisionMed, Inc., PM.

CSF donors were originally enrolled in the following studies:

1. Harvard Biomarkers Study (HBS)^14–16^: 79 HC and 43 PD were included. A subset of patients, referred to as the longitudinal cohort, (HC = 22 subjects, representing 60 samples, and PD = 18 patients, representing 45 samples) donated 2–4 CSF samples with intervals of approximately one year between each collection (See Table 1).
2. Columbia University: 17 HC and 20 PD who participated in the Biofind^17^ study at Columbia University were included. Participants were examined and donated spinal fluid in the “off” state, defined as withholding overnight dopaminergic medications.
3. PrecisionMed, Inc.: 19 HC and 18 PD CSF samples were included. Inclusion criteria for the study required at least a 1 yr PD diagnosis with stable response to dopamine therapy for >6 mo.

Sample collection for biochemical analysis was respectively approved by Partners HealthCare IRB, Columbia University IRB, and PrecisionMed IRBs, and all participants signed an informed consent. All methods were compliant with Sanofi’s guidelines and regulations.

In all cases, CSF was obtained through lumbar puncture and pooled at room temperature. Collected CSF was centrifuged at 400–2000 g for 10 min and stored at −80 °C. The time interval between collection and storage varied between CSF source (HBS: <2 h; CU: <15 min; PM: <1 h).

### CSF processing

The CSF samples were thawed on ice and aliquoted prior to use. CSF protein concentrations were determined with a Micro BCA Protein assay kit (Pierce). Approximately 20 µg of total protein containing Halt^TM^ protease and phosphatase inhibitor (ThermoFisher) was subjected to acetone precipitation. The sample was reduced with dithiothreitol, alkylated with iodacetamide and digested with recombinant LysC (1:50, Promega) and trypsin (1:25, Roche Diagnostics) in 0.1% Rapigest (Waters) in 50 mM Ammonium bicarbonate. Rapigest was precipitated with 2% formic acid and removed by centrifugation. Samples were dried and resuspended in 40 µl of 3% acetonitrile and 0.1% formic acid.

### LC/MS/MS analysis

Liquid chromatography-tandem mass spectrometry (LC/MS/MS) was performed on a Q Exactive HF hybrid Quadrupole-Orbitrap mass spectrometer (ThermoFisher) interfaced with NanoAcquity (Waters). The sample was separated using a C18 trapping (2GVM Trap Symmetry C18 column, 180 µm × 20 mm, Waters) and reverse phase column (1.8 µm HSS T3 nanoACQUITY column, 100 µm × 100 mm, Waters) for DDA and DIA acquisition over a 60 min. gradient. MS/MS spectra were acquired with top 20 ions for DDA mode with MS1 resolution of 120,000 (automatic gain control (AGC) = 3e6) and MS2 resolution of 15,000 (AGC = 1e5). An inclusion list containing 17 precursors ranging from *m/z* 400 to 1000 with varying isolation windows was used for DIA with a resolution of 60,000 (AGC = 3e6).

### DIA-MS data processing for library generation

Raw DDA files were processed in Proteome discoverer 1.4 (Thermo). Peptide identification was performed using Mascot v2.4 (Matrix Science Ltd) search against the UniProt human database (www.uniprot.org) with peptide mass tolerance of 10 ppm and fragment ion tolerance of 20 mmu. Carbamidomethyl (C) was included as a fixed modification and oxidation (M), deamidation (N,Q), phosphorylation (S,T), glutamine to pyroglutamate (N-term), acetyl (N-term), and oxidation (H,W) were included as variable modifications. The output file was imported into Spectronaut to generate the library with a maximum missed cleavage of 2, peptide length of 6 to 47 amino acid residues.

### DIA-MS data processing for CSF sample analysis

Samples were processed in Spectronaut Pulsar v11 utilizing the aforementioned library. Peptide precursor identification was set with q-value cutoff of 0.01, corresponding to a false discovery rate (FDR) of 1%. Endogenous peptides were used for retention time calibration across samples. Intensities of the top 3 peptide precursors identified for each protein were averaged, when available, to generate the protein level quantification. A local normalization approach was employed that incorporates local regression with locally weighted smoothing as previously described^13^. The variability of the distribution of protein intensities amongst samples is reduced following normalization (Additional File 1: Fig. S2). A QC standard that was processed repeatedly on multiple days and with different batches of CSF demonstrates that 191 proteins have a CV < 20% for protein quantification (Additional File 1: Fig. S3), with an overall median CV of 18%.

### Statistical analysis of cohorts for differentiating PD from HC

For univariate analysis to identify p-value, odds ratio, and AUC (area under the receiver operating characteristic (ROC) curve), a logistic regression model was fitted for each individual protein. False discovery rate^18^ is applied to adjust the multiplicity. The response variable was the binary indicator of PD status. Model covariates consisted of individual proteins. The cohorts were analyzed separately. For peptide quantification of granins, a two-tailed t-test was preformed assuming unequal variance.

### Statistical analysis of cohorts for biomarker signature development

Using Cohort 1 as the training dataset, 5 proteins identified by LASSO (least absolute shrinkage and selection operator) regression were used to build a predictive model using a multivariate logistic model to develop a biomarker signature. LASSO is a penalized regression method for selecting import variables and providing importance index for the variables^19^. Only proteins that were quantifiable in at least 90% of samples were included in the analysis (Additional File 2 and 3). The predictive model from Cohort 1 was applied to Cohort 2 for evaluation. R 3.4 software was used for the statistical analysis.

For detailed methods see Additional File 4.

### Ethics approval and consent to participate

Sample collection for biochemical analysis was approved by IRBs at Partners HealthCare, Columbia, and PrecisionMed (Protocols 7800 and 1009). All participants signed informed consents.

## Results

### Quantitative proteomics reveals unique biomarkers in PD

**Figure 1:**
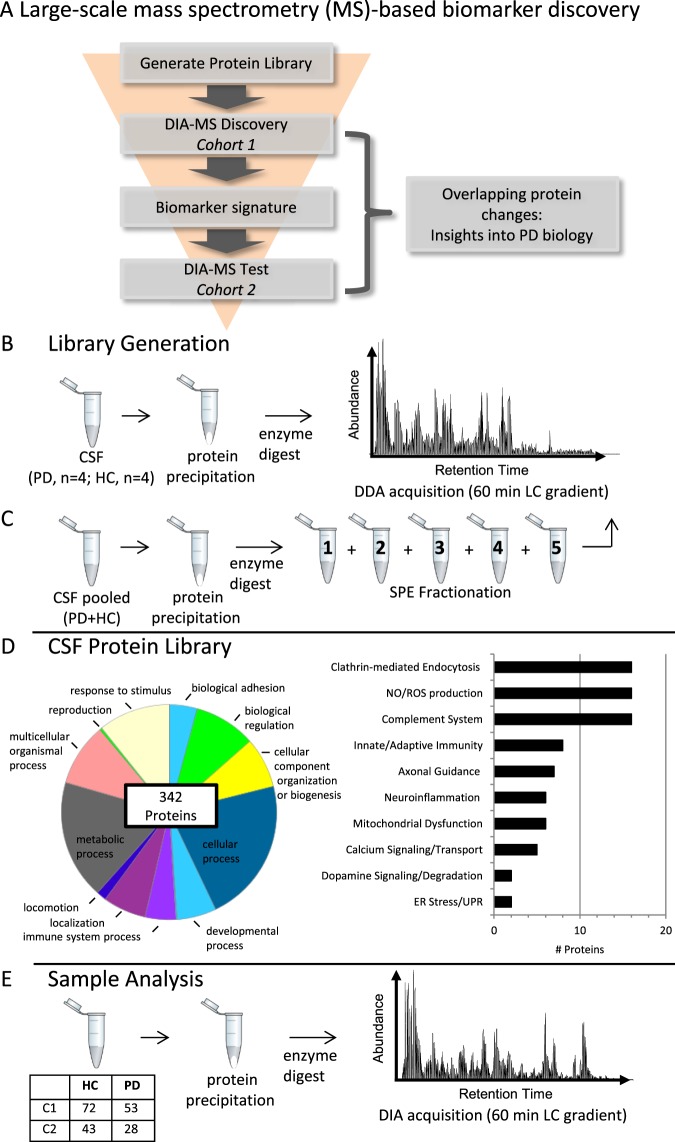
Large-scale mass spectrometry-based biomarker discovery. (**a**) DIA-MS approach overview. (**b**–**e**) Library generation and data acquisition workflow. To generate the library for processing samples by DIA-MS, a representative sample subset (**b**) and fractionated CSF pool (**c**) were processed and run in DDA-mode. (**d**) The gene ontology (go)-term bioprocess (left) classification (pantherdb.org)^20,21^ of the protein library consisting of 341 quantifiable protein groups. Proteins involved in pathways implicated in PD are indicated on the right. (**e**) CSF samples (see Table 1) were processed and analyzed by DIA-MS. A representative total ion chromatogram is shown on right. SPE = solid phase extraction, DDA = data-dependent acquisition.

To identify novel PD-relevant protein changes in CSF and unique biomarker signatures, we employed a DIA-MS workflow (Fig. 1a**)**. DIA-MS requires a peptide ion library to process and interpret complex spectra acquired. To develop a comprehensive peptide library, a subset of CSF samples were processed and analyzed by LC/MS/MS in DDA mode (see methods, Fig. 1b,c). The resulting library consisted of 2632 unique peptides, representing 341 quantifiable protein groups (Fig. 1d)^20,21^. Of note, several proteins representing PD-related pathways were included in the protein library, such as neuroinflammation and mitochondrial dysfunction (Fig. 1d, right). The limitations of the mass spectrometry approach employed in this study precluded the detection of several known PD-relevant proteins such as α-synuclein, tau, and neurofilament which have been extensively quantified in multiple cohorts^22,23^. As expected with the low volumes of CSF used in this analysis (15–50 µl/sample), known exosome associated proteins, such as flotillin-1 (FLOT1) and tumor susceptibility gene (TSG101), were not detected. The absence of these proteins suggests that the exosomal proteome has minimal contribution to the protein quantification described in this study^24^. An initial “discovery” cohort (Cohort 1) of 72 HC and 53 PD samples was analyzed by DIA-MS (Fig. 1e, Table 1). Quantification of 341 protein groups in the discovery set identified 53 proteins that were differentiated between PD and HC with a p < 0.05 by logistic regression (Additional File 2). A second “test” cohort (Cohort 2) consisting of 43 HC and 28 PD identified 41 proteins with p < 0.05 (Additional File 3). Univariate analysis and logistic regression of all 341 protein groups identified 14 proteins with an absolute odds ratio > 2 or < 0.5, and AUC > 0.6 in both cohorts (Additional File 1: Fig. S4, Additional File 5). The best performing single protein using these criteria was apolipoprotein D (APOD) with an AUC of 0.68 and 0.69 in the ROC analysis for Cohort 1 and 2, respectively (Additional File 1: Fig. S4**)**. Interestingly, APOD has also been shown to be increased in plasma of PD patients^25^.

**Figure 2:**
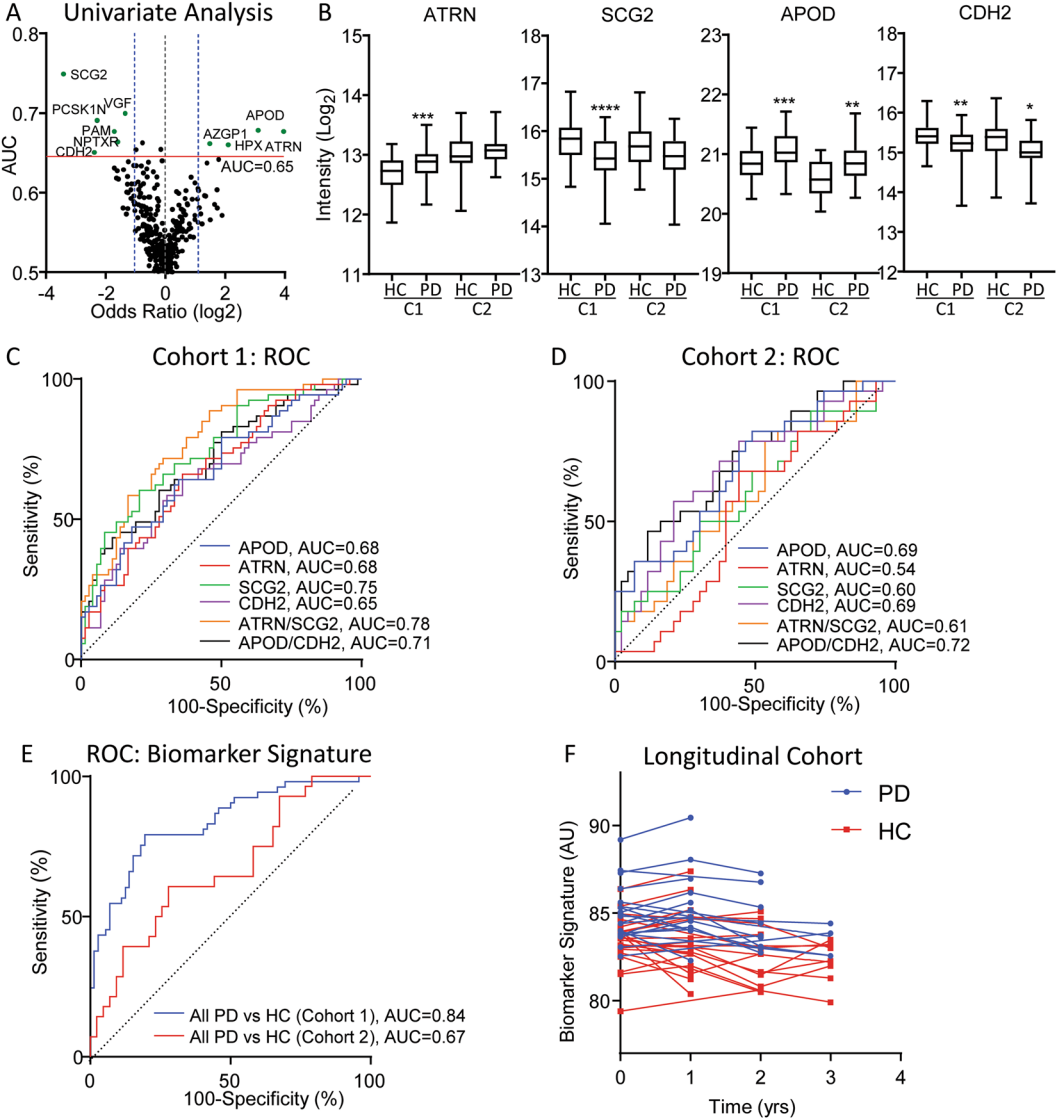
Univariate and multivariate analyses identify a PD-specific biomarker signature. (**a**) The univariate analyses of Cohort 1. Proteins highlighted in green and labeled by gene name have an AUC > 0.65 (red line) and an odds ratio (OR) > 2 or <0.5 (blue lines). (**b**) Whisker plots of the protein abundance of the top 4 predictive proteins. Significant protein changes of PD relative to the HC group are shown (Cohort 1 = C1; Cohort 2 = C2). (**c**,**d**) ROC analysis of the top proteins and protein ratios identified by Cohort 1. (**e**) ROC analysis of the predictiv**e** biomarker signature, (2.7(ATRN) + 1.2(C1QC) + 1.7(APOD)-2.2(SCG2) + 1.5(FBLN1)). (**f**) The calculated value of the biomarker signature as applied to the longitudinal cohort. (*p < 0.05; **p < 0.01; ***p < 0.001; ****p < 0.0001).

While APOD demonstrated relatively high specificity and sensitivity for a single analyte, alternative differences in disease and control groups could account for this, requiring a more robust normalization. To eliminate this potential confounding factor, we explored the predictive value of protein ratios. Using Cohort 1 as a discovery cohort, APOD and attractin (ATRN) were identified as the top 2 increasing proteins and secretogranin-2 (SCG2) and cadherin-2 (CDH2) as the top 2 decreasing proteins based on an AUC > 0.65 and the best odds ratio from the univariate analysis (Fig. 2a,b). The protein ratios of ATRN/SCG2 and APOD/CDH2 were obtained with the improved AUC of 0.78 and 0.71 in Cohort 1, respectively. However, the ATRN/SCG2 protein ratio had limited predictive value in Cohort 2 (AUC = 0.61), demonstrating a lack of consistency between PD populations. The second protein ratio, APOD/CDH2 maintained high predictive values for Cohort 2 with an AUC of 0.72, demonstrating a better predictive value over APOD alone (Fig. 2c,d).

To identify a more advanced biomarker signature that incorporates multiple proteins for increased predictive value, we performed LASSO regression and multivariate analysis on Cohort 1. A biomarker signature consisting of 5 proteins (APOD, SCG2, complement C1q subcomponent subunit C (C1QC), ATRN, and fibulin-1 (FBLN1)) was identified that separated PD from HC with an AUC of 0.84 (Fig. 2e). When the biomarker signature was applied to Cohort 2, ROC analysis determined an AUC of 0.67. The reduced predictive value in the test cohort may be due, in part, to the heterogeneity of the PD population. When we apply the biomarker signature to a longitudinal cohort consisting of 22 HC and 18 PD with repeat yearly visits, consistent elevation in PD compared to HC is observed (Fig. 2f). Larger longitudinal analyses of fast and slow progressing PD populations are required to establish whether these could be effective biomarker candidates.

### Protein changes implicate the extended granin family, cell adhesion and insulin regulation in PD

An additional strength of DIA-MS analysis, and alternative quantitative proteomics strategies, is the ability to identify protein changes that may provide insight into PD disease mechanisms. The limited number of quantifiable proteins in the CSF (341 protein groups, Fig. 1) prompted a combined pathway analysis with significant proteins identified from both Cohort 1 and 2 (p < 0.05; 95 total) pooled together. The gene ontology bioprocess classification distribution was similar to the protein library for this subset of proteins (Additional File 1: Fig. S5a,b)^20,21^. Using the CSF protein library (Fig. 1d) as the background proteome, enrichment (p < 0.08) was observed for three bioprocesses, brain development, adherens junction organization, and homophilic cell adhesion via plasma membrane adhesion molecules (Additional File 1: Fig. S5c**)**^26,27^.

**Figure 3:**
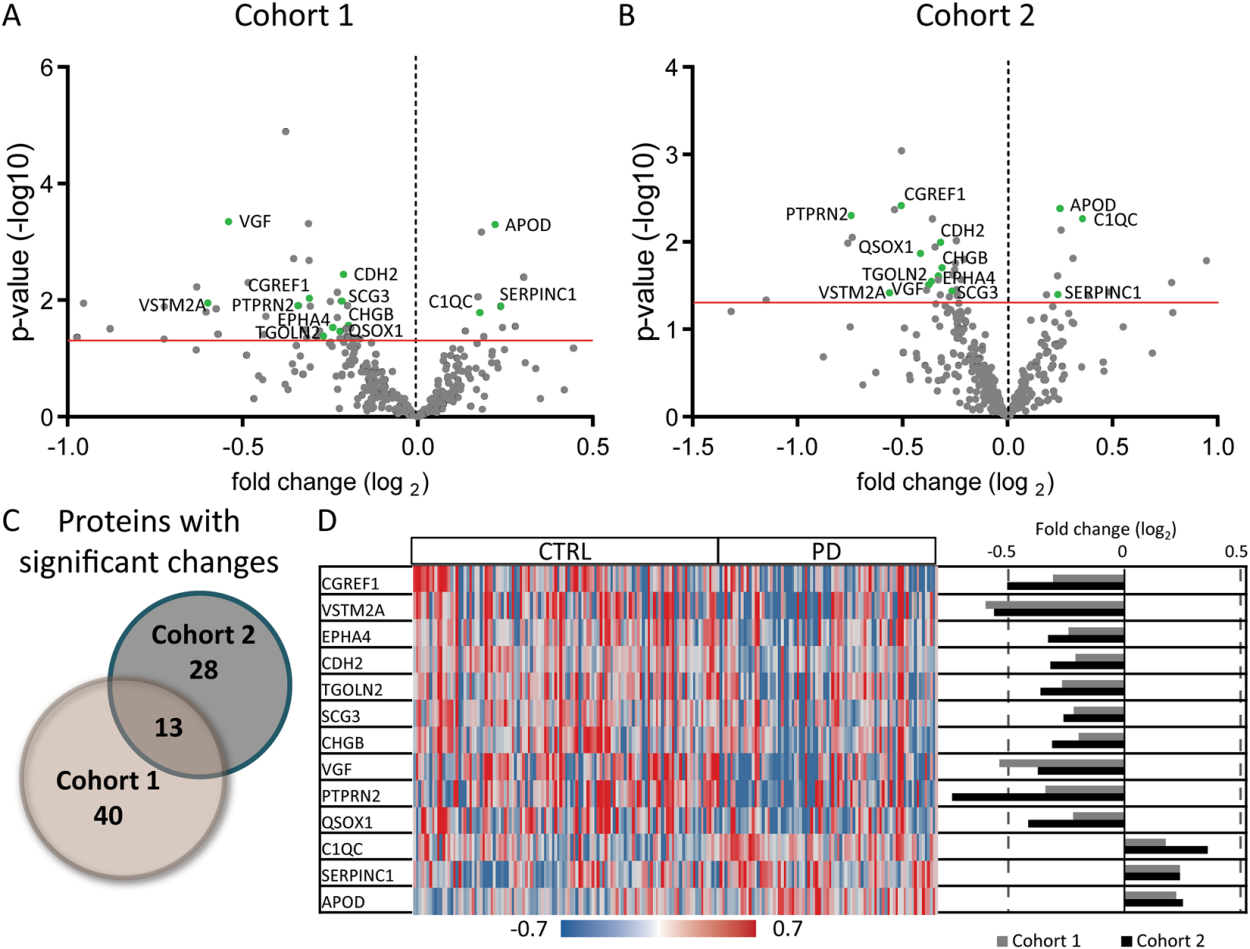
Thirteen proteins significantly change in both Cohort 1 and Cohort 2 in PD relative to HC. (**a**,**b**) Volcano plots of all proteins analyzed by DIA-MS for Cohort 1 (**a**) and Cohort 2 (**b**). Significant proteins identified in both cohorts are highlighted in green and labeled by gene name. The red line indicates a p-value cutoff of 0.05. (**c**) A Venn diagram displaying number of proteins with significant changes in Cohort 1 and 2. (**d**) Average fold change (right) compared to HC or individual sample (left) fold change normalized to mean for the 13 proteins (Top13) that were found to change in both cohorts by DIA-MS analysis in PD.

A total of 13 proteins (Top13) were found to change with a nominal p < 0.05 consistently in both cohorts (Fig. 3a,b, highlighted in green; Additional Files 2, 3 and 5), 8 of which overlap with the 14 proteins described above that demonstrated the best predictive value in univariate analysis based on absolute odds ratio and AUC. Cohort 1 had a greater number of participants with cognitive impairment (MMSE < 22: Cohort 1 = 7; Cohort 2 = 0) and a higher average disease duration than Cohort 2 (Table 1), which might contribute to the small number of overlapping significant protein changes between cohorts. The age discrepancy between PD and HC in Cohort 2 may also contribute to the small number of overlapping protein changes observed in both cohorts. Three protein levels (APOD, antithrombin-III (SERPINC1), and C1QC) increased in PD CSF compared to HC; and the remaining 10 decreased (Fig. 3c,d). Significant elevation of SERPINC1 in CSF of PD patients was confirmed by ELISA analysis of both cohorts with a fold change of 1.3 and 1.4 for Cohort 1 and 2, respectively (p < 0.05, Additional File 1: Fig. S6).

The most enriched bioprocess in the Top13 is cell adhesion, including 3 proteins, CDH2, cell growth regulator with EF hand domain protein 1 (CGREF1), and ephrin type-A receptor 4 (EPHA4). CDH2 is directly involved in cell to cell adhesion by integrating into cellular membranes and forming a “reverse zipper” with other CDH2 proteins on adjacent cells^28^. CDH2 requires removal of its propeptide domain (aa 26–159) to be functionally active as a cell adhesion molecule^29^. Based on DIA-MS analysis, 7 peptides were identified in the small propeptide domain and zero peptides in the remainder of the protein which is over 700 amino acids long with over 50 basic residues available for enzymatic cleavage (Additional File 1: Fig. S7a). In agreement with these data, quantification by ELISA of the mature CDH2 (aa 160–906), which lacks the propeptide region, failed to detect any protein in CSF (Additional File 1: Fig. S7b,c). These data suggest the cleaved propeptide region is the only portion of CDH2 present in CSF. The propeptide of CDH2 was decreased in PD compared to HC in both cohorts, suggesting a reduction in CDH2 processing, and therefore a decreased level of functionally active CDH2 in PD. In addition, several studies have failed to identify changes in total CDH2 protein in post-mortem brain tissue, consistent with results in this study which suggest that a processing deficit, rather than change in total protein level, is altered in PD^30,31^.

Another Top13 protein found to decrease in PD relative to HC, receptor-type tyrosine-protein phosphatase N2 (PTPRN2), also known as phogrin and IA-2β, is targeted to secretory granules^32^. PTPRN2 is a known regulator of insulin secretion^33,34^, and is implicated in type 1 diabetes^35^. In the present study, PTPRN2 is decreased in PD relative to HC, which may contribute to the link between insulin resistance and PD observed in certain patients^36^.

Three proteins found to decrease in PD relative to HC in both cohorts, neurosecretory protein VGF (VGF), secretogranin-3 (SCG3), and chromogranin-B (CHGB), are members of the extended granin family^37^. The granin family consists of 8 precursor proteins in total (VGF, SCG2, CHGB, SCG3, neuroendocrine protein 7B2 (SCG5), ProSAAS (PCSK1N), chromogranin-A (CHGA), neuroendocrine secretory protein 55 (GNAS)), all of which are known to be cleaved into functional peptides that undergo regulated secretion^37,38^. Additionally, CHGA is significantly reduced in Cohort 2 (p = 0.040). Moreover, in Cohort 1, which has almost double the number of samples than Cohort 2, three additional granin family members, SCG2, PCSK1N, and SCG5, are reduced in PD relative to HC. The remaining family member, GNAS, was not detectable in CSF in our study (Additional Files 2 and 3).

**Figure 4:**
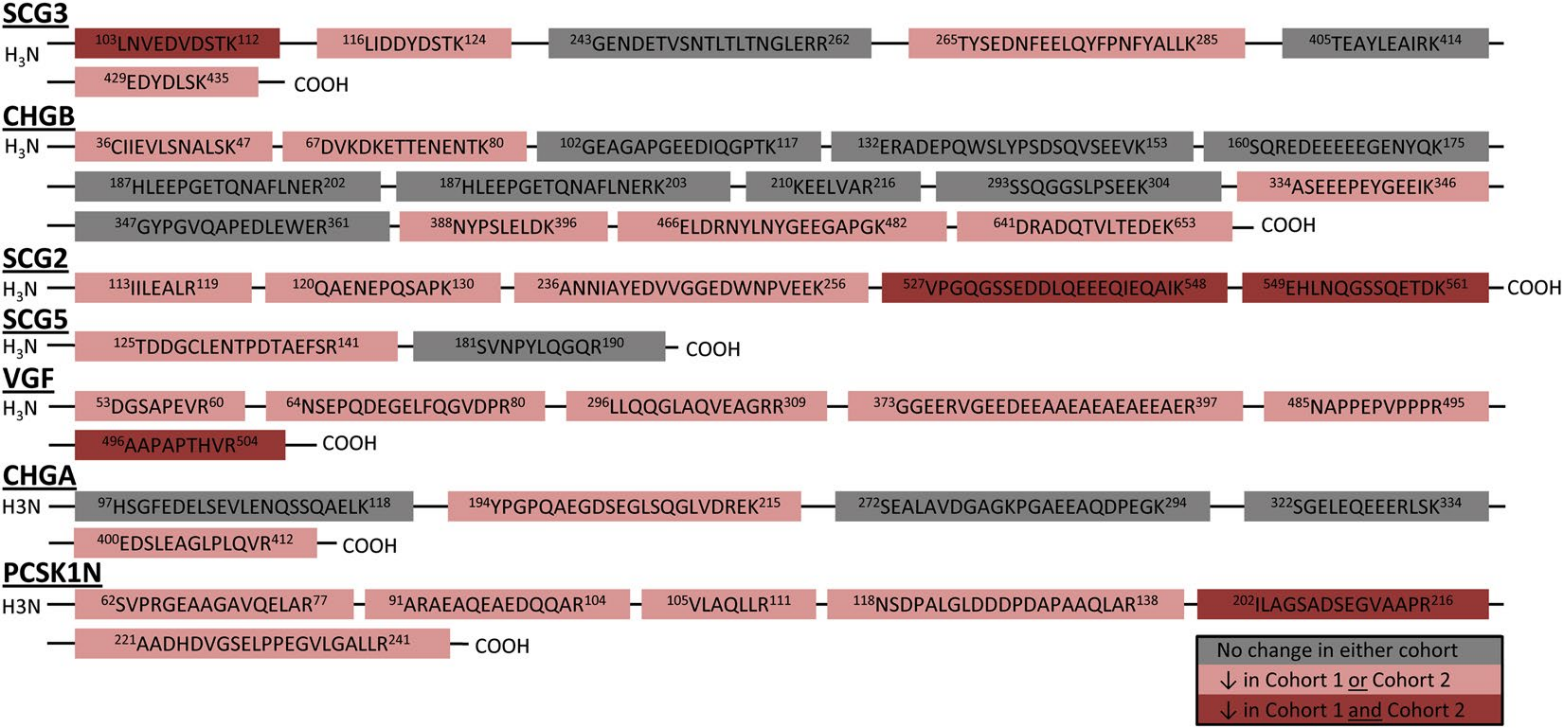
Peptide quantification reveals region-specific reduction of the extended granin family in PD. Granin peptides were found to either decrease in PD in both cohorts (red), one cohort (light red), or neither cohort (gray). The C-terminal region of PCSK1N, SCG2, and VGF are decreased in both PD cohorts relative to HC. For SCG3, the N-terminal region is decreased in PD.

The granin family members are known to be cleaved into bioactive peptides. To identify any region-specific and/or alterations in known bioactive peptides derived from the granin family members, we performed peptide level quantification of the 7 out of 8 granins that were detectable in the CSF. Only peptides that passed the manual inspection of spectra with an average q < 0.005 were considered in the analysis. All 7 proteins had peptides that were significantly decreased in PD relative to HC in at least one cohort (Fig. 4, Additional File 6). SCG2, a granin family member found to significantly decrease only in Cohort 1 at the protein level, has two C-terminal region peptides that are significantly decreased in both cohorts in the peptide level analysis. This region corresponds to the bioactive peptide manserin, which is known to be present in the neuroendocrine system and cerebellum. Additionally, several peptides do not change between PD and HC in either cohort suggesting that the total protein level of this extended granin family is unchanged, but processing, localization and/or secretion of these peptides is likely affected. Although further investigation is required to confirm this hypothesis, there is additional evidence for altered peptide processing in PD (see discussion).

## Discussion

The present study employed DIA-MS proteomics to identify protein changes in PD CSF. This quantitative approach allowed the identification of unique alterations of several biomarker candidates (as protein ratios or detection signatures). Analyses of significant protein changes implicated the granin family, peptide processing, insulin regulation and cell adhesion in PD.

The findings presented in this study suggest an altered granin family metabolism in PD. Numerous studies have implicated a subset of peptides derived from the granin family in neurodegenerative diseases^33,39–45^, the present study expands on these observations, associating nearly the entire extended granin family with PD. We observed the reduction of multiple granins that function as biologically active peptides in PD relative to HC (i.e., VGF, CHGA, CHGB, SCG2, SCG3, SCG5, and PCSK1N). The granin family plays an essential role in the regulated secretory pathway that is responsible for controlled delivery of peptides and neurotransmitters^37^. Importantly, granin peptides and catecholamines are co-stored in dense-core vesicles and granins can regulate levels and functions of catecholamines^46–48^, such as dopamine which is reduced in PD. The reduction of granin-derived biologically active peptides observed in this study might have pathophysiological implications and supports the larger catecholaminergic deficit in PD. Correspondingly, alterations in granin levels or metabolism have been reported in neurotoxin-induced models of PD^42–44^.

Alterations in specific aspects of granin biology have been reported in the context of multiple neurodegenerative diseases. Region-specific mislocalization of peptide fragments of the granin family of proteins to pathological inclusions have been observed in brain tissue from pathologically confirmed AD, PD, and Pick’s disease^39,41,49^. Deposition of alpha-synuclein, the pathological hallmark of PD, was recently found on secretogranin II-positive vesicular membranes, suggesting a mechanistic link between the reduction of granins in CSF and PD pathogenesis^50^. Previous studies have also reported distinct metabolic processing of specific granins in CSF. The N-terminal region of VGF was decreased in frontotemporal dementia whereas its C-terminal region is reduced in AD^40,45^. Interestingly, the C-terminal region of VGF was decreased in the CSF of PD patients in both cohorts in this study.

Further mechanistic support to the extensive misprocessing of granin peptides is provided by the reduction in peptidyl-glycine alpha-amidating monooxygenase (PAM). Activation of approximately half of bioactive peptides, including the granins, is mediated by PAM, a C-terminal alpha-amidating enzyme. PAM levels were reduced in PD relative to HC in Cohort 1 (p = 0.002) and in the less severely affected Cohort 2 (p = 0.066), further implicating altered precursor processing in PD. PAM is located in secretory granules with VGF, which contains a canonical PAM C-terminal alpha-amidation site. A bioactive peptide of CHGA (namely PST) requires PAM-mediated C-terminal amidation for activation^37^. The decrease in the propeptide region of CDH2 in the present study, further implicates altered peptide processing in the context of PD. Lack of CDH2 processing is known to reduce cell-to-cell adhesion efficiency^28,51^ which might in turn contribute to increased blood-brain barrier permeability.

The protein changes identified in this study provide additional support to the impaired insulin regulation in PD, including in PD dementia^36^. “Anti-insulin resistance” therapies are under investigation as therapeutic strategies for PD^52,53^. In the present study, both PTPRN2 and CHGA are decreased in PD relative to HC. Both PTPRN2, as well as the CHGA derived peptide, PST, are known modulators of insulin secretion^54^, providing a potential mechanistic explanation to the insulin resistance in PD^36,52,53^. Evaluation of larger clinically defined cohorts would be required to define the association of particular protein aberrations with specific disease phenotypes.

Bioprocess enrichment analysis identified adherens junction organization as the most over-represented biological process, representing a total of 6 proteins that were decreased in PD relative to HC: cadherins 2, 6, 8, 10 and 13 (CDH2, CDH6, CDH 8, CDH10 and CDH 13), and cell adhesion molecule 2 (CADM2). Adherens junction, along with gap and tight junctions, play a key role in the maintenance and permeability of the blood brain barrier^55^. In accord with the present study, system biology approaches have also identified alterations in the adherens junction in PD^56–58^, which might be related to the blood brain barrier compromise in PD^59,60^.

There are several limitations associated with this study that require further exploration. First, over 90% of PD patients were prescribed levodopa and/or a dopamine agonist and their contribution to the protein changes described herein has not been assessed, requiring a *de novo* PD sample set for evaluation. Second, although the standardization in DIA-MS provides acceptable peptide quantification for the peptides present in the protein library, it is quite possible that enhancing the protein library with in-depth fractionation of CSF may translate to more quantifiable proteins in the analysis of the CSF sample cohorts. Quantifying the protein changes identified by an orthogonal method would be required to confirm the findings identified in this study as well. We were able to confirm SERPINC1 elevation in PD CSF by ELISA, but were unable to extend this analysis to the granin family due to limited availability of samples and suitable antibodies specific to the identified peptides. Lastly, an independent CSF cohort would be required to determine the robustness of the protein changes observed in the longitudinal cohort.

## Conclusion

The present study identifies potential PD biomarkers and novel protein changes implicating cell adhesion, insulin regulation, and bioactive peptide processing. Importantly, the extended granin family was reduced in disease, suggesting a potential common mechanism for the biological reduction in monoamine neurotransmission in Parkinson’s patients. With several promising disease-modifying therapeutics under development^3,4^, there is an increased need for objective biomarkers to evaluate their efficacy. The continuous development of robust platforms to quantify small changes in proteins in biospecimens as well as the evaluation of larger longitudinal cohorts are critical for future patient subtyping and evaluation of therapeutics.

## Supplementary information

Additional File 1 Supplemental Figures.

Additional File 2 Table S1.

Additional File 3 Table S2.

Additional File 4 Supplemental Methods.

Additional File 5 Table S3.

Additional File 6 Table S4.

## Data Availability

All output files (.raw), search file (.sne), Swiss-prot export used in library and sample processing (.fasta), spectral library (.kit), and protein quantification data (.xlsx) have been deposited to PRIDE under the accession number: PXD011216.
