# Supplementary material for: Cerebrospinal fluid proteomics implicates the granin family in Parkinson’s disease
Source: Sci Rep. 2020 Feb 12;10:2479. doi: 10.1038/s41598-020-59414-4 (PMC7015906; doi:10.1038/s41598-020-59414-4)
Supplement: Supplementary file 1 — Additional File 1 Supplemental Figures. [file 41598_2020_59414_MOESM1_ESM.pdf]

# Additional File 1: Supplemental Figures

**Cerebrospinal fluid proteomics implicates the granin family in Parkinson's disease**

[1,2]Melissa S. Rotunno, [2]Monica Lane, [3]Wenfei Zhang, [2*]Pavlina Wolf, [2#]Petra Oliva, [1]Catherine Viel, [6]Anne-Marie Wills, [5]Roy N. Alcalay, [4,6,7]Clemens R. Scherzer, [1]Lamya S. Shihabuddin, [2*]Kate Zhang, [1]S. Pablo Sardi

[1]Rare and Neurologic Diseases Therapeutic Area, Sanofi, Inc., Framingham, MA 01701

[2]Biomarkers and Bioanalytics, Translational Sciences, Sanofi, Inc., Framingham, MA 01701

[3]Translational Medicine, Sanofi, Inc., Framingham, MA 01701

[4]Precision Neurology Program, Harvard Medical School, Brigham & Women's Hospital, Boston, MA 02115, USA

[5]Department of Neurology, Columbia University, New York, NY 10032-3784

[6]Department of Neurology, Massachusetts General Hospital, Boston, MA 02114, USA

[7]APDA Advanced Center for Parkinson's Disease Research, Harvard Medical School, Brigham & Women's Hospital, Boston, MA 02115, USA

[*]current address: Editas Medicine, Cambridge, MA 02141

[#]current address: ARCHIMED Life Sciences GmbH, Leberstraße 20/2, 1110 Vienna, Austria, Europe

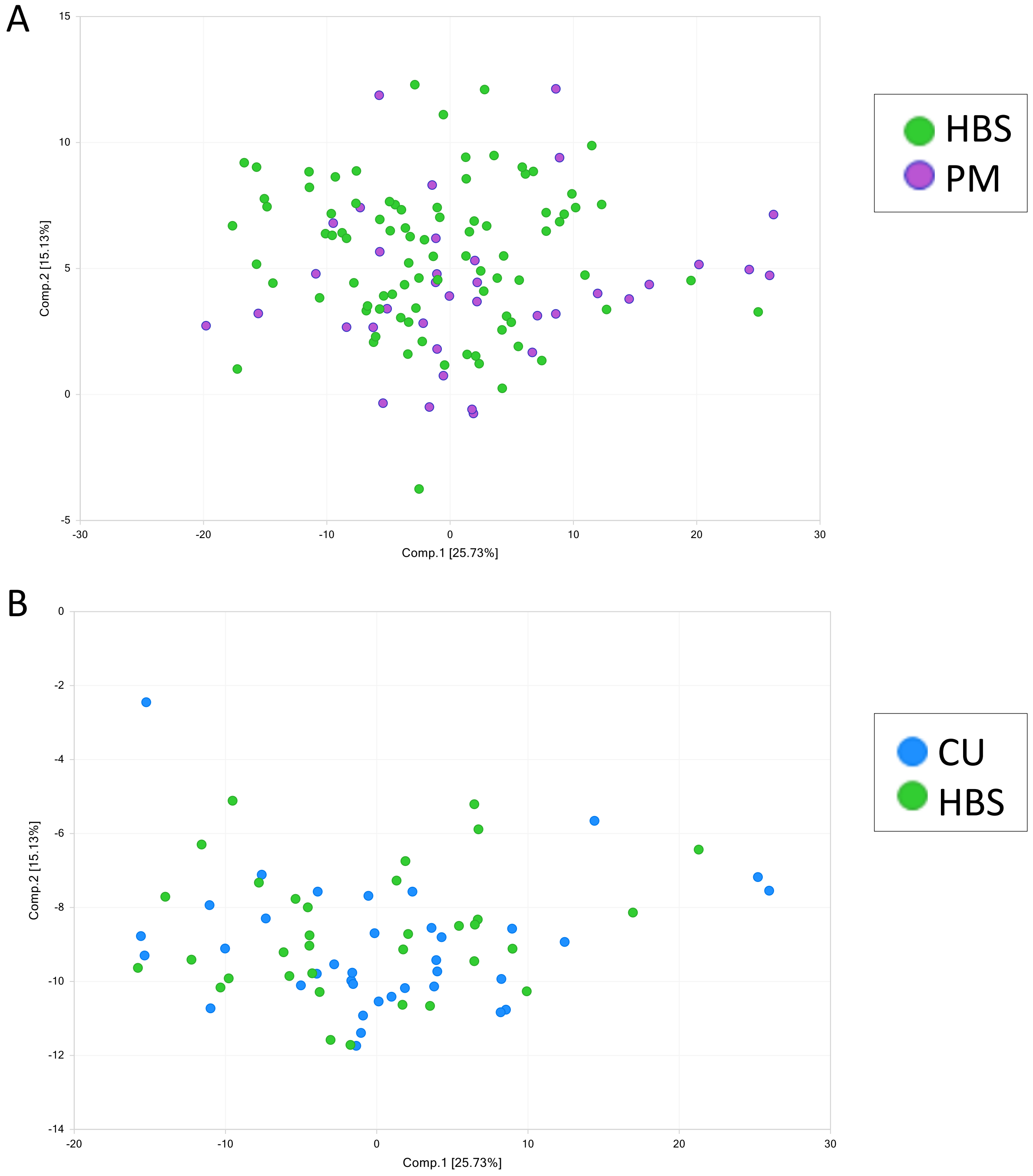


**Figure S1. Principal component analysis illustrates no observed batch effect based on CSF source**. All samples from Cohort 1 (A) and Cohort 2 (B) were subjected to principal component analysis on the $Log_2$ transformed DIA dataset. No group separation based on CSF source was observed for either cohort. Harvard biomarkers study, HBS (green); Columbia University, CU (blud); PrecisionMed, Inc., PM (purple).

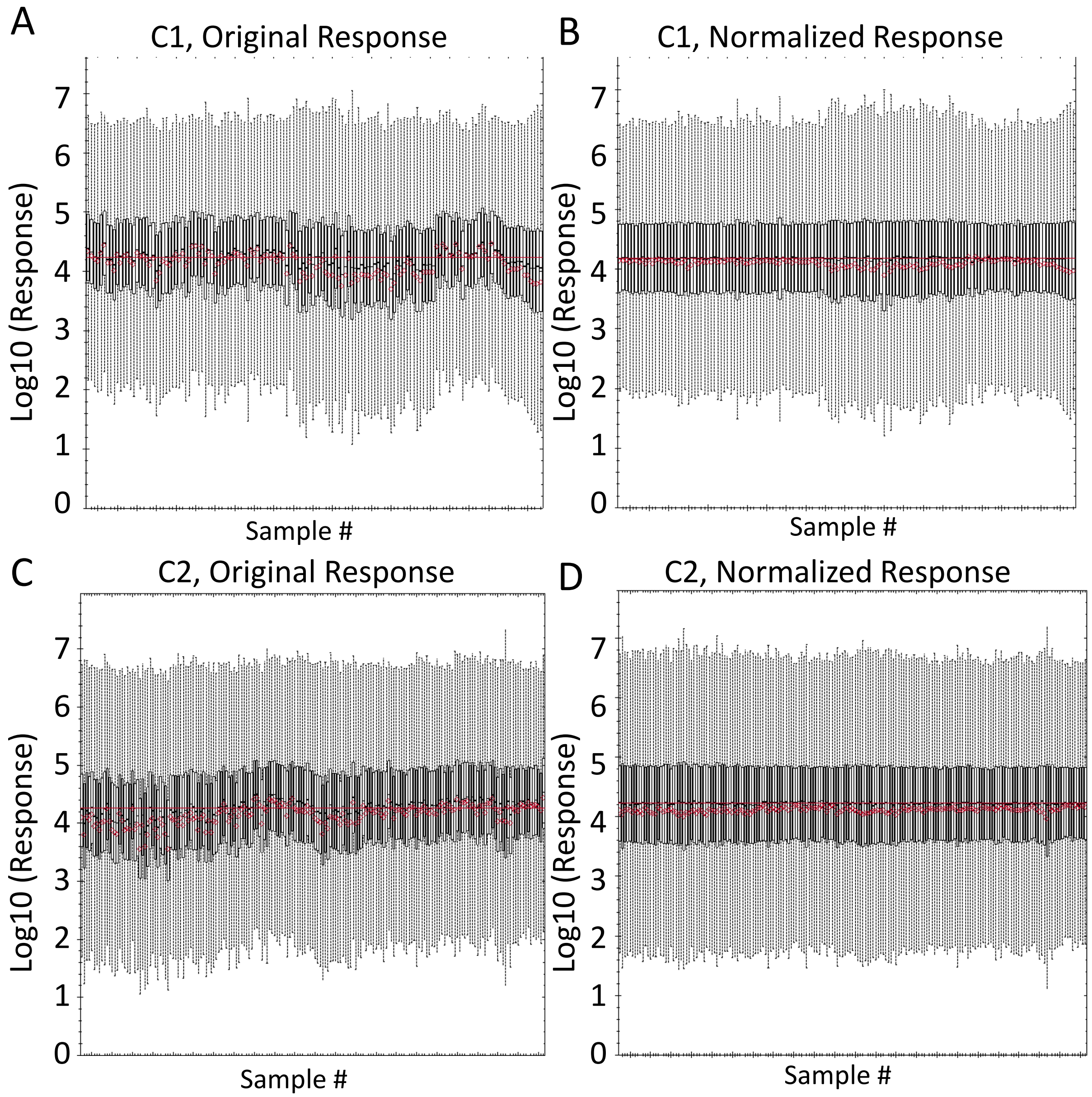


**Figure S2. Protein sample intensity distribution before and after normalization**. A and C represent the original protein intensity distribution prior to incorporating the local normalization strategy for both cohort 1 and 2, respectively. B and D illustrate the protein intensity distribution following normalization for cohort 1 and 2, respectively.

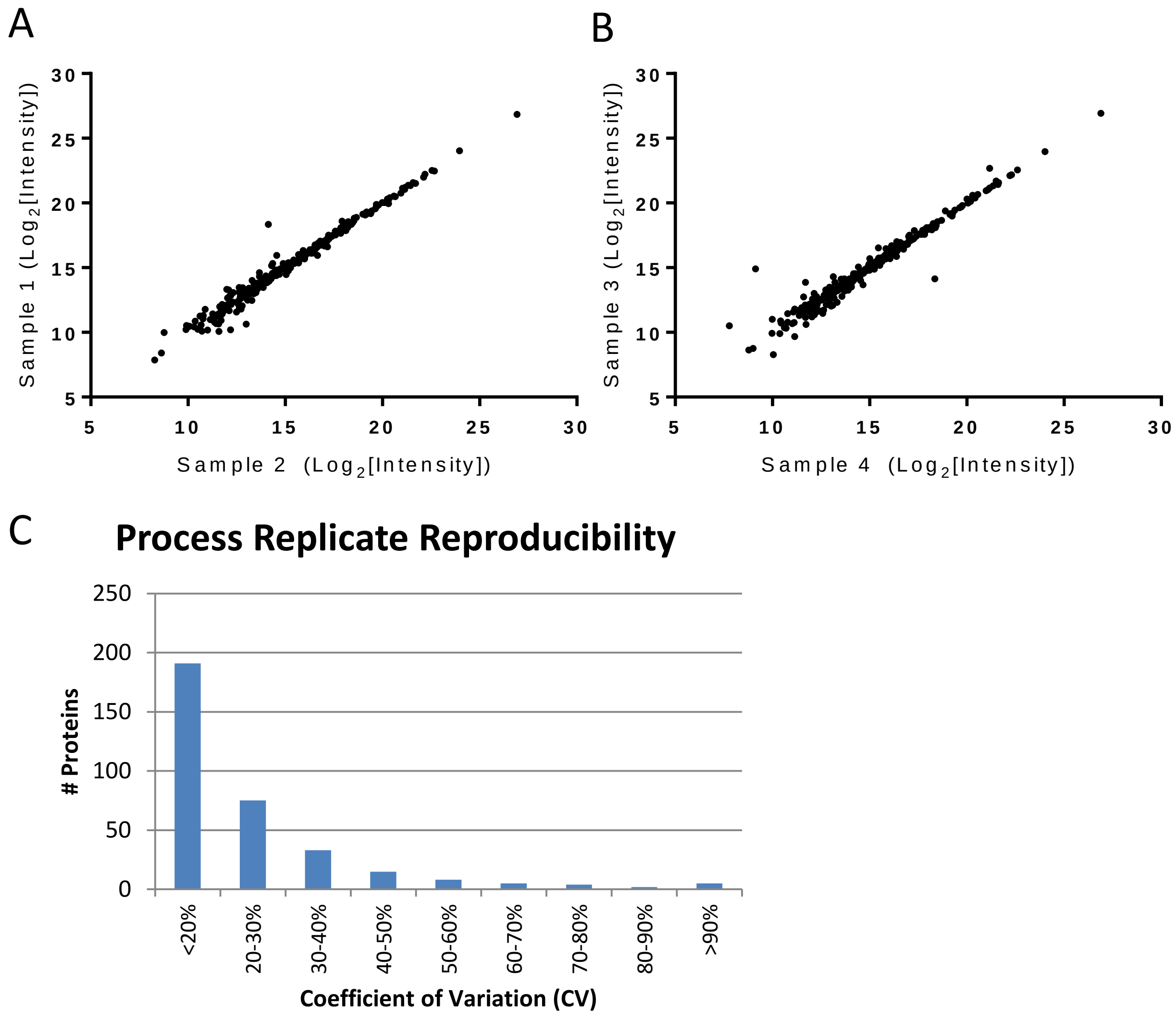


**Figure S3. Reproducibility of QC standard CSF processing**. The QC standard was processed ten times over the course of several weeks to generate process replicates for assessing the reproducibility of the CSF processing with acetone precipitation. (**A**,**B**) Representative side-by-side comparison of the protein intensities for two process replicates. (**C**) Summary of the distribution of the coefficient of variation (CV) of the QC standard process replicates.

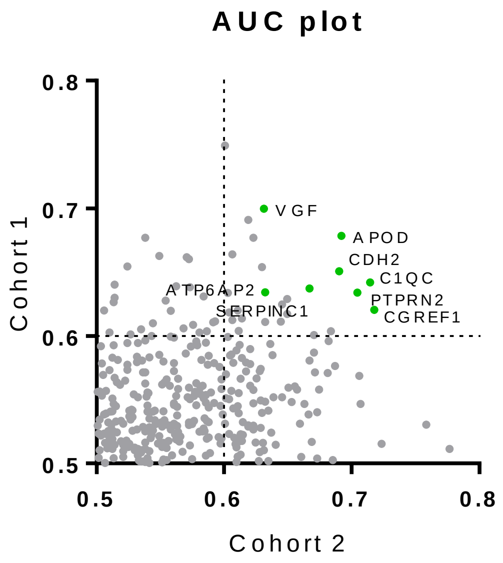


**Figure S4. Comparison of AUC values between cohort 1 and 2**. Area under the curve (AUC) values were calculated from the ROC curves of the univariate analysis. Proteins with AUC>0.63 in both cohorts are highlighted in green.

A

B

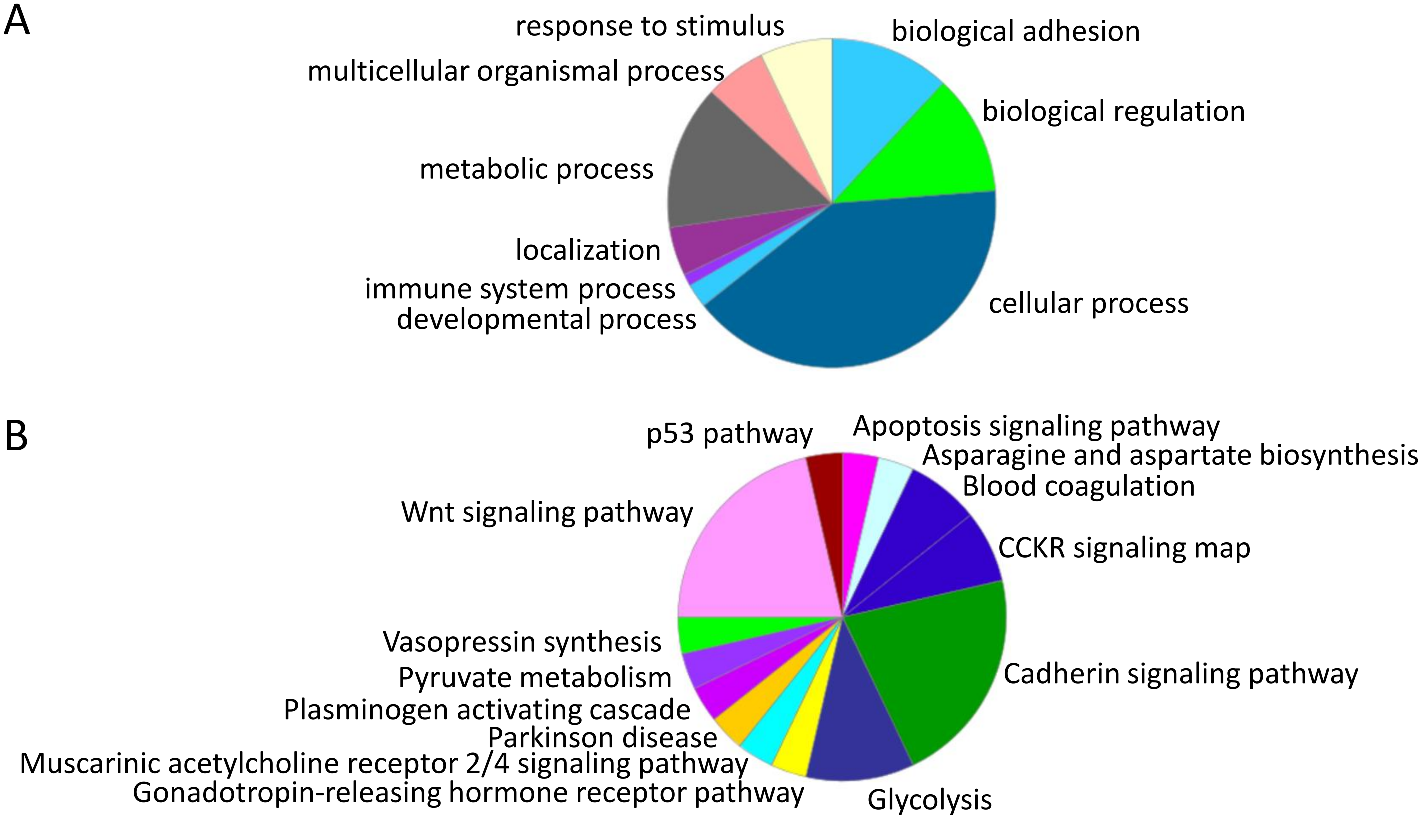


C

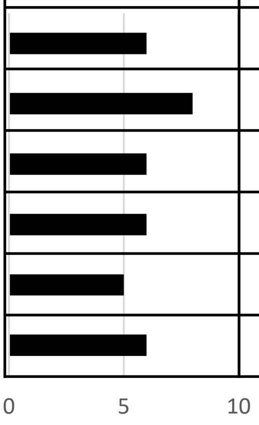


| | # Proteins | Category | p-value |
|---|---|---|---|
| adherens junction organization | | GOTERM_BP_DIRECT | 0.015 |
| homophilic cell adhesion via plasma membrane adhesion molecules | | GOTERM_BP_DIRECT | 0.028 |
| Cadherin conserved site | | INTERPRO | 0.032 |
| Cadherin | | INTERPRO | 0.053 |
| brain development | | GOTERM_BP_DIRECT | 0.076 |
| Cadherin-like | | INTERPRO | 0.079 |

**Fig. S5. Summary of Pathway Analysis for Significant Proteins Identified in Cohort 1 and 2 combined.** (A)The gene ontology (go)-term bioprocess classification (PANTHER14.1) of the significant protein changes identified in Cohort 1 and 2. (B) A summary of the PANTHER Pathways identified using the significant protein changes identified in Cohort 1 and 2. (C) Pathway enrichment analysis using DAVID Bioinformatics Resources 6.8. The proteins in the CSF protein library (Fig. 1) was used as the background proteome.

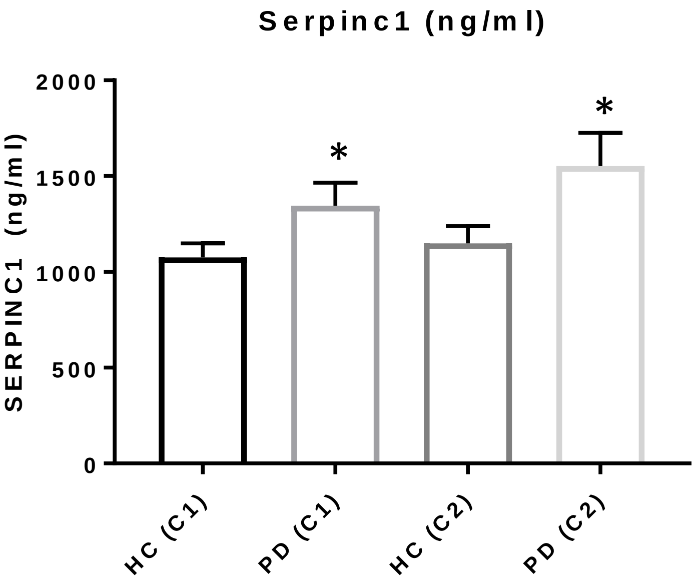


**Figure S6. SERPINC1 levels elevated in CSF of PD patients as quantified by ELISA.** All CSF samples from cohort 1 and 2 were analyzed by SERPINC1 ELISA (LSBio, #LS-F10410) at a 1:2000 dilution in technical replicate. In both cohorts, SERPINC1 was elevated in PD relative to HC, according to a one-tailed student's t-test assuming unequal variance. (*, p-value<0.05)

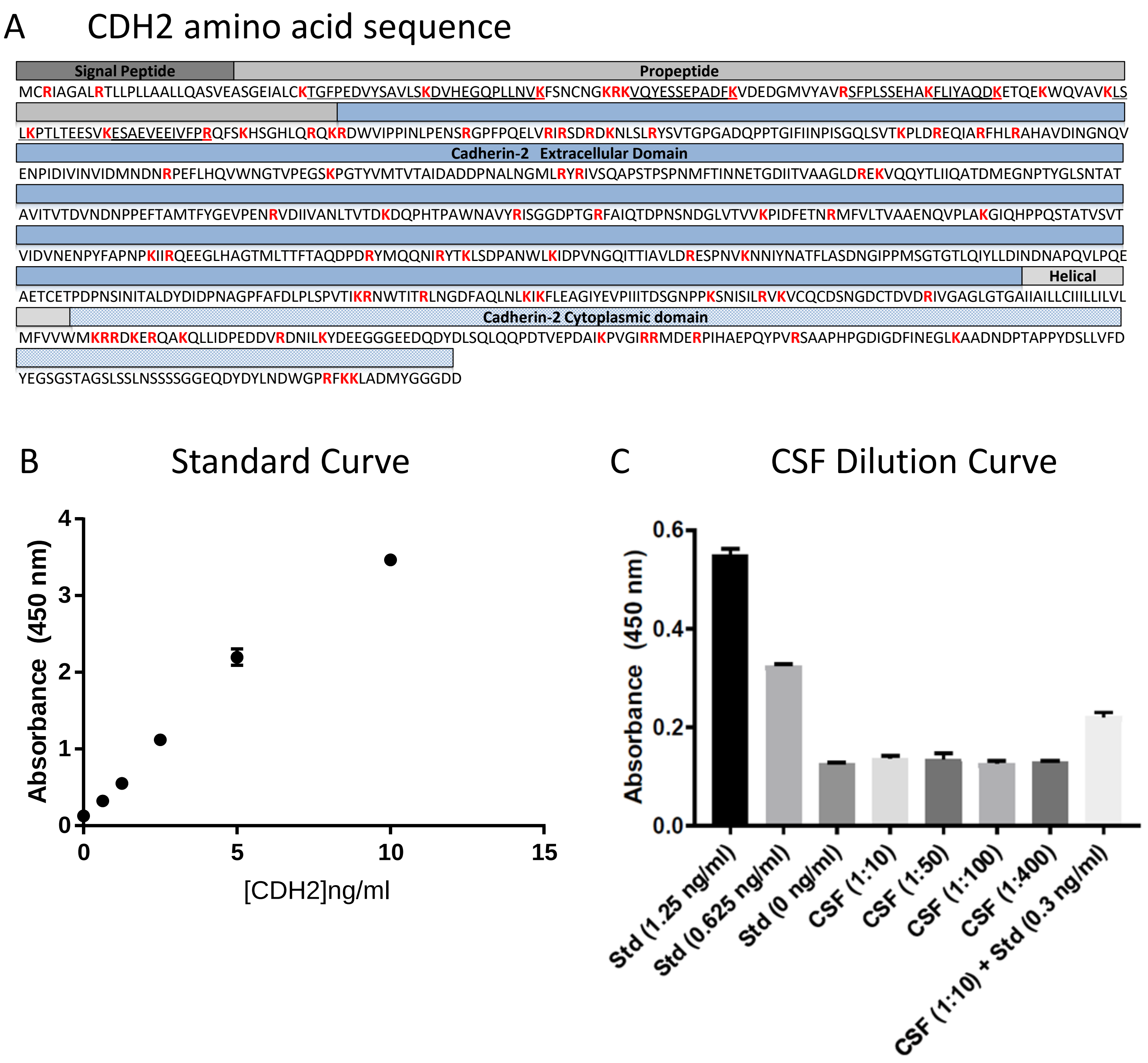


**Figure S7. Reduction in the propeptide region of n-cadherin in PD.** (**A**) Sequence coverage of the CDH2 protein. The CDH2 protein sequence is shown as well as the known domains. Underlined residues indicate sequence coverage in mass spectrometry and were only identified in the propeptide region of CDH2. Available lysines and arginines for digestion with LysC and trypsin are shown in red. (**B-C**) ELISA for the mature CDH2 protein (lacking the propeptide region) detects CDH2 at low concentrations using the standard. (**C**) Dilution series of CSF pool (PD and HC) does not show any signal above the buffer blank (Std 0). When low concentrations of CDH2 standard were spiked into pooled CSF, the ELISA was able to detect the protein.
