# Supplementary material for: Cerebrospinal fluid proteomics implicates the granin family in Parkinson’s disease
Source: Sci Rep. 2020 Feb 12;10:2479. doi: 10.1038/s41598-020-59414-4 (PMC7015906; doi:10.1038/s41598-020-59414-4)
Supplement: Supplementary file 4 — Additional File 4 Supplemental Methods. [file 41598_2020_59414_MOESM4_ESM.docx]

**Additional File 4: Supplemental Methods**

**Cerebrospinal fluid proteomics implicates the granin family in Parkinson’s disease**

^1,2^Melissa S. Rotunno, ^2^Monica Lane, ^3^Wenfei Zhang, ^2*^Pavlina Wolf, ^2#^Petra Oliva, ^1^Catherine Viel, ^6^Anne-Marie Wills, ^5^Roy N. Alcalay, ^4,6,7^Clemens R. Scherzer, ^1^Lamya S. Shihabuddin, ^2*^Kate Zhang, ^1^S. Pablo Sardi

^1^Rare and Neurologic Diseases Therapeutic Area, Sanofi, Inc., Framingham, MA 01701

^2^Biomarkers and Bioanalytics, Translational Sciences, Sanofi, Inc., Framingham, MA 01701

^3^Translational Medicine, Sanofi, Inc., Framingham, MA 01701

^4^Precision Neurology Program, Harvard Medical School, Brigham & Women's Hospital, Boston, MA 02115, USA

^5^Department of Neurology, Columbia University, New York, NY 10032-3784

^6^Department of Neurology, Massachusetts General Hospital, Boston, MA 02114, USA

^7^APDA Advanced Center for Parkinson's Disease Research, Harvard Medical School, Brigham & Women's Hospital, Boston, MA 02115, USA

^*^current address: Editas Medicine, Cambridge, MA 02141

^#^current address: ARCHIMED Life Sciences GmbH, Leberstraße 20/2, 1110 Vienna, Austria, Europe

**Supplemental Methods**

**Cohort distribution of CSF samples.** Cohort 1, comprised of CSF samples from PrecisionMed, Inc. and Harvard Biomarkers Study, was used as the training dataset for the biomarker signature development. Cohort 2, comprised of CSF samples from Columbia University and Harvard Biomarkers Study, was used as the evaluation dataset. To identify disease relevant protein changes, significant proteins identified in both Cohort 1 and 2 independently were studied in more detail (See **Table 1** for more details).

**CSF processing**. CSF protein concentrations were determined with a Micro BCA Protein assay kit (Pierce). A total of 20 µg of protein containing Halt^TM^ protease and phosphatase inhibitor (Thermo) was diluted to 200 µl with HPLC grade water followed with 800 µl of cold acetone. Samples were incubated at 4°C for 1 h and centrifuged at 13,000 rpm for 20 min at 4°C. Supernatants were discarded and the pellets were washed twice with 1 mL of cold acetone. The pellets were dried down in a SpeedVac (ThermoFisher) and resuspended in 25 µl 0.1% Rapigest (Waters) in 50 mM Ammonium bicarbonate with 10 mM dithiothreitol and incubated at 65°C for 1 h. Following reduction, the samples were alkylated with 2.6 µl of 200 mM iodacetamide and incubated at room temperature for 1 h in the dark. Samples were then digested with 0.4 µg recombinant LysC (1:50, Promega) at 37°C for 16 h followed by 0.8 µg of trypsin (1:25, Roche Diagnostics) for 2.5 h at 37°C. Samples were incubated at 37°C for 45 min with 2% formic acid to precipitate Rapigest . Rapigest was then removed by centrifugation. Supernatant was transferred to a new Eppendorf tube and were dried and resuspended in 40 µl of 3% acetonitrile and 0.1% formic acid prior to MS analysis. A total of 2 µl of each sample was injected into the instrument for LC-MS/MS analysis. To generate the quality control (QC) standard, 15 HC from PrecisionMed, Inc were pooled based on CSF volume. To generate the CSF pool for fractionation and building the peptide ion library, 2 PD CSF samples from PrecisionMed, Inc. were added at equal protein amount to the QC standard to obtain 60 µg of total protein (HC = 30 µg, 33.1 µl;PD = 30 µg, 25.8 µl). The CSF pool was digested as described above, except that the dried peptides were resuspended in 20 mM Ammonium formate at pH 10 and fractionated. Peptides were fractionated by binding to a HLB 96-well sorbent plate (Cat#186001828BA, Waters) at 10 µg/well and eluting with increasing acetonitrile (11%, 14.5%, 17.5%, 21%, and 45%) concentration in 20 mM Ammonium formate.

**LC/MS/MS analysis**. Liquid chromatography-tandem mass spectrometry (LC/MS/MS) was performed on a Q Exactive HF hybrid Quadrupole-orbitrap mass spectrometry (ThermoFisher) interfaced with NanoAcquity (Waters). The sample was separated using a C18 trapping (2GVM Trap Symmetry C18 column, 180 µm x 20mm, Waters) and reverse phase column (1.8 µm HSS T3 nanoACQUITY column, 100 µm x 100mm, Waters) for DDA and DIA acquisition over a 60 min gradient (see below for details).

| Liquid chromatography gradient for both DIA and DDA sample acquisition methods | | | |
| --- | --- | --- | --- |
| Time (min) | Flow rate (µl/min) | 0.1% Formic Acid (%) | Acetonitrile, 0.1% Formic acid (%) |
| 0 | 0.50 | 97 | 3 |
| 4 | 0.50 | 90.1 | 9.9 |
| 32 | 0.50 | 82.9 | 17.1 |
| 48 | 0.50 | 73.9 | 26.1 |
| 56 | 0.50 | 59.5 | 40.5 |
| 60 | 0.50 | 15 | 85 |
| 62 | 0.75 | 15 | 85 |
| 72 | 0.75 | 15 | 85 |
| 73 | 0.75 | 97 | 3 |
| 77 | 0.50 | 97 | 3 |
| 85 | 0.5 | 97 | 3 |

MS/MS spectra were acquired with top 20 ions for DDA mode with MS1 resolution of 120,000 (automatic gain control (AGC) = 3e6), injection time of 30 ms, and a scan range of 375 to 1600 m/z. MS2 was acquired with a resolution of 15,000 (AGC = 1e5), injection time of 50 ms, isolation window of 1.5 m/z, and normalized collision energy (NCE) of 29. The spray voltage was set to 2500 and the capillary temperature set to 275°C. For DIA-MS, MS1 was collected at a resolution of 60,000 with a maximum injection time of 30 ms and an AGC target of 3e6. MS2 was acquired with a resolution of 60,000, an AGC target of 3e6, and a maximum injection time of 120 ms. An inclusion list containing 17 precursors ranging from m/z 400 to 1000 with varying isolation windows was used (See table below for more detail). All CSF samples were randomized prior to data acquisition.

| DIA Precursors | |
| --- | --- |
| m/z | Isolation window (m/z) |
| 413 | 26 |
| 437.5 | 27 |
| 462.5 | 27 |
| 487.5 | 27 |
| 512.5 | 27 |
| 537.5 | 27 |
| 562.5 | 27 |
| 587.5 | 27 |
| 615 | 32 |
| 645 | 32 |
| 675 | 32 |
| 705 | 32 |
| 745 | 52 |
| 795 | 52 |
| 845 | 52 |
| 895 | 52 |
| 959.5 | 81 |

**DIA-MS data processing for library generation**. The raw DDA files of 5 CSF pool fractions, (as described in “CSF processing”) and 8 unfractionated CSF samples (PD=4, HC=4) were combined and processed in Proteome discoverer 1.4 (Thermo). Peptide identification was performed using Mascot v2.4 (Matrix Science Ltd) search against the uniprot human database ([www.uniprot.org](http://www.uniprot.org)) with peptide mass tolerance of 10 ppm and fragment ion tolerance of 20 mmu. The human protein database file used in the search was exported on 7/27/2017 from Swiss-Prot with isoforms and only reviewed entries included. Additionally, the common repository of adventitious proteins (cRAP) from The Global Proteome Machine ([www.thegpm.org](http://www.thegpm.org)) were included for a total of 42,272 entries. Carbamidomethyl (C) was included as a fixed modification and oxidation (M), deamidation (N,Q), phosphorylation (S,T), glutamine to pyroglutamate (N-term), acetyl (N-term), and oxidation (H,W) were included as variable modifications. The output file was imported into Spectronaut to generate the library with a maximum missed cleavage of 2, peptide length of 6 to 47 amino acid residues.

**DIA-MS data processing for CSF protein quantification.**

Peptide level quantification reflects the sum of the fragment ions that are present across samples. For protein quantification, Spectronaut employs the strategy reported in Silva et al., 2006: “Absolute quantification of proteins by LCMSE: a virtue of parallel MS acquisition” Mol Cell Proteomics 5(1): 144-56.” This method determined that the three most intense tryptic peptides for given protein analyzed in a label-free proteomics experiment produced an average signal response that correlates with the protein’s molar concentration.  In Spectronaut’s approach, the software ranks the peptide precursors meeting the FDR cutoff (q<0.01) in order of highest area intensity to lowest when taking mean response across all replicates in the experiment. The three most intense precursors detectable in all samples were then averaged to determine the protein’s relative abundance.  Where less than 3 peptides were detected for a given protein, relative abundance is determined by average of the N peptides detected (i.e. 1 or 2). For peptide level quantification of granin proteins, an average q-value of <0.005 was required for inclusion in analyses. Spectra were also manually inspected to ensure overlapping transitions and defined peaks above the noise.

All output files (.raw), search file (.sne), Swiss-prot export used in library and sample processing (.fasta), spectral library (.kit), and protein quantification data (.xlsx) have been deposited to PRIDE under the accession number: PXD011216.

**ELISA.** SERPINC1 (LSBio, # LS-F10410) and CDH2 (LSBio, #LS-F4699) ELISA’s were performed according to the manufacturer’s protocol. For SERPINC1, CSF was diluted 1:2000 prior to addition to the antibody-coated plate. For CDH2, CSF was diluted to 1:10, 1:50, 1:100, and 1:400. All samples were processed in technical replicate.
