# Supplementary material for: Cerebrospinal fluid proteomics implicates the granin family in Parkinson’s disease
Source: Sci Rep. 2020 Feb 12;10:2479. doi: 10.1038/s41598-020-59414-4 (PMC7015906; doi:10.1038/s41598-020-59414-4)
Supplement: Supplementary file 6 — Additional File 6 Table S4. [file 41598_2020_59414_MOESM6_ESM.docx]

**Additional File 6: Table S4 Peptide level changes of the extended granin family**

**Cerebrospinal fluid proteomics implicates the granin family in Parkinson’s disease**

^1,2^Melissa S. Rotunno, ^2^Monica Lane, ^3^Wenfei Zhang, ^2*^Pavlina Wolf, ^2#^Petra Oliva, ^1^Catherine Viel, ^6^Anne-Marie Wills, ^5^Roy N. Alcalay, ^4,6,7^Clemens R. Scherzer, ^1^Lamya S. Shihabuddin, ^2*^Kate Zhang, ^1^S. Pablo Sardi

^1^Rare and Neurologic Diseases Therapeutic Area, Sanofi, Inc., Framingham, MA 01701

^2^Biomarkers and Bioanalytics, Translational Sciences, Sanofi, Inc., Framingham, MA 01701

^3^Translational Medicine, Sanofi, Inc., Framingham, MA 01701

^4^Precision Neurology Program, Harvard Medical School, Brigham & Women's Hospital, Boston, MA 02115, USA

^5^Department of Neurology, Columbia University, New York, NY 10032-3784

^6^Department of Neurology, Massachusetts General Hospital, Boston, MA 02114, USA

^7^APDA Advanced Center for Parkinson's Disease Research, Harvard Medical School, Brigham & Women's Hospital, Boston, MA 02115, USA

^*^current address: Editas Medicine, Cambridge, MA 02141

^#^current address: ARCHIMED Life Sciences GmbH, Leberstraße 20/2, 1110 Vienna, Austria, Europe

**Table S4. Peptide level changes of the extended granin family**

|  | | **Used for DIA Quantification** | |  | **fold change (PD/CTRL)** | | **p-value**** | |
| --- | --- | --- | --- | --- | --- | --- | --- | --- |
| **Gene Name** | **Peptide** | **C1** | **C2** | **Q value (avg)** | **C1** | **C2** | **C1** | **C2** |
| **SCG3** | ^103^LNVEDVDSTK^112^ | Yes | Yes | 1.5E-03 | 0.86 | 0.81 | 1.5E-02 | 1.2E-02 |
|  | ^116^LIDDYDSTK^124^ | Yes | Yes | 1.4E-03 | 0.89 | 0.87 | 4.7E-02 | 7.6E-02 |
|  | ^243^GENDETVSNTLTLTNGLERR^262^ | No | No | 4.3E-03 | 0.88 | 0.81 | 1.1E-01 | 2.3E-01 |
|  | ^265^TYSEDNFEELQYFPNFYALLK^285^ | No | No | 3.0E-03 | 0.96 | 0.82 | 7.3E-01 | 2.3E-02 |
|  | ^405^TEAYLEAIRK^414^ | Yes | Yes | 1.7E-03 | 0.89 | 0.94 | 9.6E-02 | 5.5E-01 |
|  | ^429^EDYDLSK^435^ | No | No | 4.7E-03 | 0.84 | 0.88 | 7.3E-03 | 2.1E-01 |
| **CHGB** | ^36^CIIEVLSNALSK^47^ | No | No | 3.1E-04 | 0.83 | 0.88 | 6.9E-03 | 1.7E-01 |
|  | ^67^DVKDKETTENENTK^80^ | No | No | 2.5E-03 | 0.84 | 0.79 | 1.1E-02 | 1.3E-01 |
|  | ^102^GEAGAPGEEDIQGPTK^117^ | No | No | 6.6E-04 | 0.91 | 0.85 | 2.3E-01 | 1.6E-01 |
|  | ^132^ERADEPQWSLYPSDSQVSEEVK^153^ | No | No | 3.9E-05 | 1.01 | 0.86 | 8.5E-01 | 6.9E-02 |
|  | ^160^SQREDEEEEEGENYQK^175^ | No | No | 6.1E-05 | 0.85 | 0.79 | 5.5E-02 | 8.9E-02 |
|  | ^187^HLEEPGETQNAFLNER^202^ | No | No | 2.6E-04 | 0.92 | 0.87 | 4.9E-01 | 2.1E-01 |
|  | ^187^HLEEPGETQNAFLNERK^203^ | No | No | 2.3E-04 | 0.90 | 0.85 | 1.9E-01 | 2.0E-01 |
|  | ^210^KEELVAR^216^ | No | No | 2.4E-03 | 0.94 | 0.78 | 4.5E-01 | 6.9E-02 |
|  | ^293^SSQGGSLPSEEK^304^ | Yes | Yes | 3.0E-06 | 0.90 | 0.84 | 1.3E-01 | 7.0E-02 |
|  | ^334^ASEEEPEYGEEIK^346^ | Yes | Yes | 4.1E-05 | 0.92 | 0.75 | 2.7E-01 | 3.1E-02 |
|  | ^347^GYPGVQAPEDLEWER^361^ | No | No | 3.9E-03 | 1.02 | 0.91 | 8.7E-01 | 4.9E-01 |
|  | ^388^NYPSLELDK^396^ | Yes | Yes | 4.8E-06 | 0.90 | 0.82 | 1.0E-01 | 3.7E-02 |
|  | ^466^ELDRNYLNYGEEGAPGK^482^ | No | No | 9.8E-06 | 0.89 | 0.80 | 7.9E-02 | 4.6E-02 |
|  | ^641^DRADQTVLTEDEK^653^ | No | No | 7.3E-04 | 0.82 | 0.87 | 2.3E-02 | 1.3E-01 |
| **SCG2** | ^113^IILEALR^119^ | Yes | Yes | 5.4E-06 | 0.72 | 0.97 | 4.0E-05 | 7.5E-01 |
|  | ^120^QAENEPQSAPK^130^ | No | No | 2.6E-03 | 0.66 | 0.97 | 2.3E-07 | 7.3E-01 |
|  | ^236^ANNIAYEDVVGGEDWNPVEEK^256^ | No | Yes | 1.6E-03 | 0.93 | 0.79 | 4.7E-01 | 3.7E-02 |
|  | ^527^VPGQGSSEDDLQEEEQIEQAIK^548^ | No | No | 2.1E-03 | 0.80 | 0.76 | 6.5E-03 | 2.1E-02 |
|  | ^549^EHLNQGSSQETDK^561^ | Yes | Yes | 5.8E-06 | 0.82 | 0.81 | 4.1E-04 | 4.7E-03 |
| **SCG5** | ^125^TDDGCLENTPDTAEFSR^141^ | Yes | Yes | 7.0E-04 | 0.68 | 0.99 | 5.7E-04 | 8.9E-01 |
|  | ^181^SVNPYLQGQR^190^ | Yes | Yes | 8.6E-04 | 0.86 | 0.86 | 1.1E-01 | 9.7E-02 |
| **VGF** | ^53^DGSAPEVR^60^ | Yes | No | 2.2E-03 | 0.79 | 0.82 | 4.7E-02 | 1.3E-01 |
|  | ^64^NSEPQDEGELFQGVDPR^80^ | No | Yes | 7.6E-04 | 0.67 | 0.86 | 5.9E-05 | 1.8E-01 |
|  | ^296^LLQQGLAQVEAGRR^309^ | No | No | 6.0E-04 | 0.46 | 1.13 | 6.2E-07 | 4.3E-01 |
|  | ^373^GGEERVGEEDEEAAEAEAEAEEAER^397^ | Yes | Yes | 1.2E-05 | 0.76 | 0.86 | 2.3E-03 | 1.9E-01 |
|  | ^485^NAPPEPVPPPR^495^ | Yes | Yes | 4.1E-04 | 0.74 | 0.80 | 3.4E-03 | 5.2E-02 |
|  | ^496^AAPAPTHVR^504^ | No | No | 1.8E-03 | 0.76 | 0.77 | 4.5E-02 | 3.1E-02 |
| **CHGA** | ^97^HSGFEDELSEVLENQSSQAELK^118^ | Yes | Yes | 5.6E-06 | 0.90 | 0.81 | 3.6E-01 | 1.1E-01 |
|  | ^194^YPGPQAEGDSEGLSQGLVDREK^215^ | No | No | 1.1E-03 | 0.88 | 0.84 | 1.3E-01 | 3.8E-02 |
|  | ^272^SEALAVDGAGKPGAEEAQDPEGK^294^ | Yes | Yes | 1.6E-06 | 0.88 | 0.83 | 1.6E-01 | 5.4E-02 |
|  | ^322^SGELEQEEERLSK^334^ | No | Yes | 1.6E-03 | 0.88 | 0.87 | 2.2E-01 | 2.8E-01 |
|  | ^400^EDSLEAGLPLQVR^412^ | No | No | 3.5E-04 | 0.72 | 1.13 | 8.1E-03 | 4.2E-01 |
| **PCSK1N** | ^62^SVPRGEAAGAVQELAR^77^ | No | No | 6.9E-04 | 0.78 | 1.01 | 3.6E-03 | 9.6E-01 |
|  | ^91^ARAEAQEAEDQQAR^104^ | No | No | 1.3E-03 | 0.73 | 0.94 | 6.8E-04 | 5.3E-01 |
|  | ^105^VLAQLLR^111^ | Yes | Yes | 7.2E-06 | 0.75 | 0.92 | 7.1E-03 | 2.8E-01 |
|  | ^118^NSDPALGLDDDPDAPAAQLAR^138^ | No | Yes | 2.1E-06 | 0.74 | 0.89 | 1.2E-04 | 2.2E-01 |
|  | ^202^ILAGSADSEGVAAPR^216^ | Yes | Yes | 1.5E-06 | 0.77 | 0.84 | 2.4E-05 | 4.5E-02 |
|  | ^221^AADHDVGSELPPEGVLGALLR^241^ | Yes | No | 5.5E-05 | 0.92 | 0.72 | 2.9E-01 | 5.2E-03 |

*red values: p-value<0.05, as determined by a two-tailed t-test assuming unequal variance
